# Resmetirom for MASH: A Comprehensive Review of a Novel Therapeutic Frontier

**DOI:** 10.3390/biomedicines13092079

**Published:** 2025-08-26

**Authors:** Angad Tiwari, Ashish Sharma, Harendra Kumar, Varnika Gupta, Vishal Deshpande, Jaya Sai Mupparaju, Tanisha Mishra, Hareesha Rishab Bharadwaj, Dushyant Singh Dahiya, Varun Jain

**Affiliations:** 1 Department of Internal Medicine, Maharani Laxmi Bai Medical College, Jhansi 284001, Uttar Pradesh, India; angadtiwari@outlook.com; 2 Department of Internal Medicine, Yale New Haven Hospital, New Haven, CT 06504, USA; 3 Department of Internal Medicine, Dow University of Health Sciences, Karachi 75200, Pakistan; 4 Suburban Community Hospital, Norristown, PA 19401, USA; 5 Department of Internal Medicine, Krishna Institute of Medical Sciences, Secunderabad 500003, Telangana, India; 6 Department of Internal Medicine, NRI Medical College, Guntur 522503, Andhra Pradesh, India; jayasaimupparaju@gmail.com; 7 Department of Internal Medicine, University of Connecticut School of Medicine, Hartford, CT 06030, USA; 8 Royal Stoke University Hospital, University Hospitals of North Midlands NHS Trust, Stoke-on-Trent ST4 6QG, Staffordshire, UK; 9 Division of Gastroenterology, Hepatology & Motility, The University of Kansas School of Medicine, Kansas City, KS 66160, USA; 10 St. Francis Hospital and Medical Center, University of Connecticut, Farmington, CT 06105, USA; varunjainafmc@gmail.com; 11 Frank H. Netter School of Medicine, Quinnipiac University, North Haven, CT 06473, USA

**Keywords:** resmetirom, MASH (metabolic dysfunction-associated steatohepatitis), metabolic syndrome, liver fibrosis, obesity, dyslipidaemia

## Abstract

Metabolic dysfunction-associated steatohepatitis (MASH) is a progressive liver disease linked to type 2 diabetes (T2D), obesity, and dyslipidaemia, which are all parts of the metabolic syndrome. In 2024, for non-cirrhotic MASH with mild to advanced fibrosis, resmetirom, a selective thyroid hormone receptor-β agonist, became the first FDA-approved treatment for this condition. By increasing β-oxidation and lipid metabolism, it minimises systemic thyroid or cardiac effects while reducing hepatic fat, inflammation, and fibrosis. Resmetirom is being developed for use in combination with lifestyle interventions, such as diet and exercise, to maximize patient benefit. Nevertheless, the lack of congruence between clinical trial populations and real-world payer criteria underscores access obstacles that necessitate policy reform. The successful delivery of screening programs depends on the education of providers from various disciplines and the establishment of uniform screening standards. Future studies should investigate the clinical application of resmetirom in combination with agents that may provide additional benefits, such as GLP-1 receptor agonists, SGLT2 inhibitors, and statins. These results are significant in light of recent long-term safety monitoring of these agents, particularly regarding the thyroid axis. Ensuring equitable uptake will be crucial, as it involves defining fair access through payer endpoints, conducting cost-effectiveness analysis, and considering patient-reported outcomes. Resmetirom represents a breakthrough in MASH management, offering potential metabolic benefits in conjunction with comprehensive clinical and lifestyle approaches.

## 1. Introduction

Metabolic dysfunction-associated steatohepatitis (MASH), previously known as non-alcoholic steatohepatitis (NASH), is a progressive liver disease characterised by hepatic steatosis, inflammation, and hepatocyte injury. MASH raises a significant health concern due to its potential for liver-related complications like fibrosis, cirrhosis, and even hepatocellular carcinoma [1]. Common risk factors associated with the development of MASH are obesity, dyslipidaemia, type 2 diabetes mellitus, and metabolic syndrome [2]. Hypothyroidism, polycystic ovary syndrome, obstructive sleep apnoea, hypopituitarism, and hypogonadism are other identified risk factors, especially in the Asia–Pacific region [3].

MASH affects a substantial portion of the population. A study performed by Younossi et al. reported that more than a quarter of the adult world population and 7–14% of children and adolescents are suffering from metabolic dysfunction-associated steatotic liver disease (MASLD), the umbrella term that includes MASH [2]. The prevalence of MASLD has increased recently. The global prevalence is reported to be about 30% [4].

The mainstay for treating MASH has been lifestyle changes, including dietary adjustments to induce a calorie deficit and regular physical exercise. Dietary changes include adopting a diet rich in fruits, complex carbohydrates, and Mediterranean-style foods, while avoiding red meat, processed foods, and added sugars. Decreasing daily calorie intake by 500–1000 calories can lead to improved insulin sensitivity, reduced liver enzyme levels, and lower liver fat content [5]. In MASLD, a 5–7% loss in body weight can reverse steatosis, and a 10% loss can help reverse fibrosis. However, maintaining this degree of weight loss is challenging, and success rates in clinical practice often fall short of those achieved in controlled trials [6,7]. Studies have shown that vitamin E results in significant improvement in serum markers of liver inflammation, such as AST and ALT, along with improved liver histology in MASH patients. However, it has not shown promising results in reducing fibrosis [8]. Bariatric surgery has been demonstrated to be an effective long-term solution for weight loss, leading to reduced cardiovascular risk and cancers in individuals with morbid obesity.

Additionally, it also improves metabolic dysfunctions, such as MASH and liver fibrosis. Consequently, current guidelines recommend bariatric surgery as a treatment option for patients with non-cirrhotic MASLD/MASH who fulfil the criteria for bariatric surgery [9]. Currently, clinical trials are being conducted on other drugs to assess their efficacy in treating MASH. Phase 3 clinical trials are underway for GLP-1 receptor agonist (semaglutide), FGF21 analogue (pegazosatermin, efruxifermin), and PPAR pan agonist (lanifibranor) [10].

In March 2024, resmetirom (Rezdiffra) was approved by the FDA as the first pharmacological treatment for adults with MASH and mild-to-moderate liver scarring (fibrosis). Resmetirom (MGL-3196) is a liver-directed, orally administered, selective thyroid hormone receptor-β agonist. It is designed to treat MASH by enhancing hepatic lipid metabolism and decreasing lipotoxicity, thereby addressing the underlying metabolic dysfunctions associated with MASH [11]. In the MAESTRO-NASH Phase 3 clinical trial, resmetirom has shown significant improvement in liver fibrosis and resolution of MASH without worsening the fibrosis [1]. Resmetirom is the first FDA-approved drug for MASH, a promising achievement in the treatment of this severe and progressive disease. We present an overview of MASH, including the recent development of resmetirom as a promising therapeutic option. In the following sections, detailing its mode of action, clinical trial results, and comparative efficacy, we aim to highlight the potential to revolutionize MASH treatment.

In this review, we synthesize the clinical development of resmetirom to date. A structured comparison of resmetirom to other therapeutic contenders, such as GLP-1 receptor agonists, FGF21 analogues, and pan PPAR agonists, has been conducted, thereby offering readers a contextualized view of its place in the evolving treatment landscape. In addition, we provide a perspective that integrates real world considerations, including guideline recommendations and patient access challenges, and also highlights the role of lifestyle interventions alongside combination strategies in clinical practice that are often missing in the literature. Overall, the timely nature and policy related aspect of this work, along with the level of detail presented comparatively, underscores its importance to clinicians and researchers traversing this rapidly changing therapeutic landscape.

## 2. Methodology

A comprehensive literature search was conducted to identify relevant studies on metabolic dysfunction-associated steatohepatitis (MASH) and the therapeutic role of resmetirom. The search strategy included major biomedical databases such as PubMed/MEDLINE, Embase, Scopus, and the Cochrane Library. Additional sources were explored using ClinicalTrials.gov to capture ongoing and completed clinical trials, and reference lists of key articles were screened to ensure completeness.

The following keywords and Medical Subject Headings (MeSH) were used in various combinations:

○ “Metabolic dysfunction-associated steatohepatitis” OR “MASH” OR “nonalcoholic steatohepatitis” OR “NASH”
○ “Resmetirom” OR “MGL-3196” OR “thyroid hormone receptor beta agonist”
○ “Metabolic syndrome”, “liver fibrosis”, “obesity”, “dyslipidaemia”

Boolean operators (AND/OR) were applied to refine searches. The time frame was January 2015 to May 2025, ensuring inclusion of the most recent clinical trials, regulatory approvals, and translational studies. Only English-language, peer-reviewed articles, clinical guidelines, and trial reports were included.

Articles were screened independently by title and abstract to determine eligibility, followed by full-text review. Data extracted from eligible studies included study design, patient population, intervention details, outcomes assessed, and safety signals. Both preclinical and clinical evidence were considered to provide a comprehensive overview.

## 3. Pathophysiology of MASH

Nonalcoholic fatty liver disease (NAFLD), or metabolic dysfunction-associated steatosis liver disease (MASLD), is a liver condition that is linked to overweight, obesity, diabetes mellitus, and metabolic syndrome [12].

Metabolic dysfunction-associated steatohepatitis (MASH), previously known as nonalcoholic steatohepatitis (NASH), is a severe form of metabolic dysfunction-associated liver disease (MASLD) characterized by steatosis, chronic hepatocellular injury, and inflammation [13]. The distinction and diagnosis of MASH is essential as it is more progressive than NAFLD. MASH is also different from other chronic liver diseases like hepatitis B, hepatitis C, and alcoholic liver disease (ALD), which link directly to the aetiology of the disease [14].

The key histopathological features of MASH include steatosis, lobular inflammation, hepatocyte ballooning, Mallory–Denk bodies (MDB), and fibrosis [14]. MDBs are irregular eosinophilic inclusion bodies that look like caterpillar-like aggregates. The presence of MDBs in MASH is less prominent than alcoholic steatohepatitis (ASH) [14]. Hepatocyte ballooning represents the ballooning degeneration of hepatocytes due to swelling and vacuolization, resulting in a loss of the regular hexagonal shape of liver lobules [14]. Ballooning degeneration and MDB represent chronic liver injury and essential for MASH diagnosis [14].

Understanding the mechanisms behind disease progression is just as crucial as making the diagnosis. If left undiagnosed or untreated, MASH can advance to fibrosis, cirrhosis, and hepatocellular carcinoma through multiple pathways. While the role of lipid accumulation in MASLD is well established, the exact triggers that drive its progression to MASH remain poorly defined despite numerous studies. Chronic liver injury or inflammation is the central mechanism driving this progression. Lipid accumulation in hepatocytes can lead to lipotoxicity, triggering cell death, inflammation, mitochondrial dysfunction, and oxidative stress [15]. Studies have identified various lipids, free fatty acids, and free cholesterol as toxic species that cause oxidative stress [16]. Hepatocyte death activates hepatic stellate cells, which differentiate into myofibroblasts and produce excessive extracellular matrix [15]. The Hedgehog pathway is reactivated during liver repair; however, its prolonged activation can lead to aberrant tissue repair and disease progression [17]. Recent research highlights the active role of the immune system in MASLD, with the complex interplay between innate and adaptive immune cells significantly contributing to MASLD pathogenesis [18]. Various immune cells, including macrophages, neutrophils, B cells, T cells, and dendritic cells, are involved in driving disease progression [19,20]. B cells, in particular, have been shown to activate other immune cells and produce pathogenic antibodies [19]. The gut microbiome also plays a crucial role in MASLD development, interacting with the immune system and influencing hepatic inflammation [21]. Dendritic cells have a dual role in lipid accumulation and triggering inflammation [22]. Understanding these immunological mechanisms is essential for developing novel therapeutic strategies, including immunomodulatory approaches and microbiome-targeted therapies [23,24].

A study was conducted to investigate whether inflammasome deficiency leads to TNF-alpha expression, which drives MASH progression. The study demonstrated that the NLRP6 and NLRP3 inflammasomes, along with IL-18, contribute to the protection against NAFLD/MASH progression and metabolic syndrome by regulating gut microbiota composition [25]. In inflammasome-deficient mouse models, altered microbiota increases the influx of TLR4 and TLR9 agonists into the portal circulation, promoting hepatic TNF-α expression and worsening steatosis and inflammation, thereby accelerating MASH progression [25]. Understanding these mechanisms is crucial for developing new therapeutic strategies that target these mechanisms to control the disease [26].

**Figure 1:**
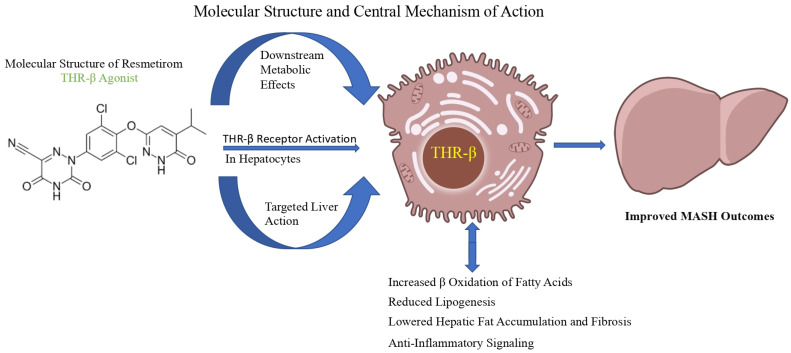
Molecular structure and central mechanisms of action of resmetirom (THR-β agonist).

**Figure 2:**
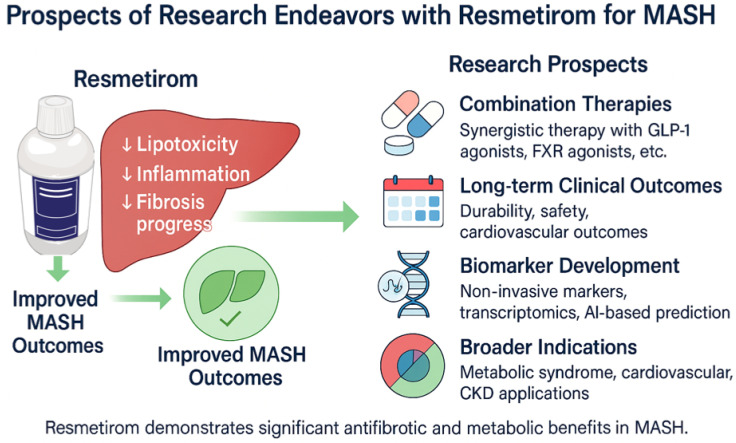
Prospects of research endeavours with resmetirom for MASH.

Several clinical trials have been designed to develop a grading system for MASH that includes the Brunt system, the NASH CRN system, and the SAF system [15]. These systems have been designed to standardize the evaluation of histological features for research and clinical purposes. However, it is still challenging to make a diagnosis among pathologists, particularly with borderline cases [14]. The molecular structure and central mechanisms of action of resmetirom are illustrated in Figure 1, while the prospective research endeavours and future directions for its application in MASH are summarized in Figure 2.

## 4. Resmetirom: Mechanism of Action and Preclinical Data

### 4.1. Preclinical Studies

Resmetirom, a selective thyroid hormone receptor-β (THR-β) agonist, demonstrated liver-specific efficacy and safety in preclinical models without impacting the central thyroid axis [27,28,29]. The rationale for targeting thyroid hormone receptors (THR) is that many large-scale epidemiological studies and meta-analyses have shown a direct link between hypothyroid state and development of MASH (previously NAFLD) [30,31]. THR-β is primarily expressed in the liver, and its activation regulates hepatic fat metabolism [27,30,32]. In pre-clinical mouse models, investigators have observed that knockdown of hepatocyte deiodinase 1 (DIO1) in diet-induced obese (DIO) mice and THR-β loss of function knock-in mice was sufficient to increase hepatic fat content [29,30,33,34,35]. Linkage studies have shown similarity in mice and humans in that humans bearing THR-B loss-of-function variants had greater hepatic fat content on ultrasound [29,36]. DIO1 or THR-B loss of function causes reduced mitochondrial lipid oxidation, decreased mitochondrial fatty acid import, and increased expression of acetyl-CoA and fatty acid synthetase, which is consistent with increased hepatic de novo lipogenesis (DNL) [29]. In diet-induced obese (DIO) mice, resmetirom reduced hepatic triglyceride (TG) accumulation, lowered cholesterol levels, and decreased liver size [27]. The compound showed a good liver-to-plasma ratio (8:1), proving liver-specific action without systemic adverse effects, which are commonly caused by non-selective thyroid hormone agonists [27]. Furthermore, resmetirom did not induce cardiac-specific α-myosin heavy chain (α-MHC) mRNA in thyroidectomized rats, affirming its lack of thyroid hormone receptor-alpha (THR-α) mediated cardiac effects [6]. In a first human study, resmetirom reduced LDL-C by up to 30% and TG by up to 60% at doses ≥50 mg/day, without suppressing thyroid-stimulating hormone (TSH) or altering free T3 levels [27,28,37]. Resmetirom’s therapeutic promise was further supported by its evaluation in a DIO mouse model with biopsy-confirmed MASH with fibrosis [38]. After 34 weeks of a high-fat, high-fructose, and high-cholesterol diet, mice treated with resmetirom (3 mg·kg^−1^ daily) for 8 weeks showed significant reductions in liver weight, hepatic steatosis, and plasma ALT/AST levels, alongside normalized plasma cholesterol and lowered fasting blood glucose—all without affecting body weight or food intake [38]. Resmetirom improved the NAFLD activity score (NAS) by decreasing steatosis and inflammation [38].

### 4.2. Mechanism of Action of Resmetirom

Resmetirom is an orally administered, selective THR-β partial agonist that predominantly targets the liver [2,39]. It has 28-fold selectivity to THR-β over THR-α and approximately 84% of the action of the natural T3 [27,39]. The reduced affinity for THR-α, which is expressed in the heart and bone, decreases the risk of cardiovascular and skeletal side effects [28,40]. It enters the hepatocytes via the organic anion transporting polypeptide 1B1 (OATP1B1) and selectively activates THR-β, which heterodimerizes with the retinoid X receptor (RXR) to regulate the transcription of genes involved in lipid metabolism, thereby mimicking the beneficial metabolic effects on endogenous T3 [27]. Upon activation, it modulates genes responsible for lipid metabolism through multiple mechanisms, ultimately downregulating hepatic fat accumulation. It increases cholesterol breakdown via activation of the hepatic enzyme, cholesterol 7-alpha-hydroxylase (CY7A1), resulting in increased LDL uptake and bile acid synthesis, resulting in lowered serum LDL levels [38,39,41]. Additionally, it inhibits sterol regulatory element-binding protein-1 (SREBP-1) and reduces de novo lipogenesis [41]. By stimulating THR-β, resmetirom stimulates autophagic processes, including lipophagy and mitophagy [41]; promotes mitochondrial biogenesis; and promotes fatty acid β-oxidation [39,41]. It also induces the expression of DIO1, which locally enhances conversion of T4 to T3 and reduces levels of inactive reverse T3 [29,41] and carnitine palmitoyltransferase 1 (CPT1), promoting oxidative phosphorylation. This helps reduce reactive oxygen species (ROS) and improve insulin sensitivity [29,39]. Additionally, resmetirom exerts anti-inflammatory and anti-fibrotic effects by suppressing inflammatory pathways such as NF-κB and Jak-STAT3 [33]. It downregulates pro-inflammatory cytokines such as TNF-α and IL-6, attenuates Kupffer cell activation, and reduces fibrogenesis by suppressing transforming growth factor-beta (TGF-β) signalling [39]. This dual action improves hepatic steatosis, inflammation, and fibrosis, as demonstrated in clinical trials [31].

In preclinical and clinical investigations, resmetirom has been shown to reduce serum triglycerides, low-density lipoprotein cholesterol (LDL-C), and apolipoprotein B (ApoB) and simultaneously raise sex hormone-binding globulin (SHBG) levels, which is a pharmacodynamic biomarker of hepatic THR-β activation [39]. Resmetirom’s SHBG-elevating effects are suggestive of broader metabolic benefits than liver-specific activities, probably enhancing insulin sensitivity and lowering diabetes risk, which warrants further research to expand the domain of the drug [39].

## 5. Clinical Trials and Results

Resmetirom’s FDA clearance on 14 March 2024, for non-cirrhotic MASH with moderate to advanced fibrosis addressed the essential lack of licensed therapies for MASH by demonstrating its considerable efficacy and safety [42], as outlined through a comparative analysis of relevant clinical trials summarized in Table 1.

### 5.1. Phase 2 Trial: MGL-3196-05 (NCT02912260)

In a 36-week, randomized, double-blind, placebo-controlled Phase 2 trial (MGL-3196-05; NCT02912260) [47] conducted at 25 centres across the USA, 125 overweight or obese adults with biopsy-confirmed MASH and fibrosis stages F1 to F3 were enrolled [11]. To be eligible participants, the hepatic fat fraction was ≥10% of the MRI proton density fat fraction (MRI-PDFF) at baseline [11]. Randomisation was 2:1, and participants were administered either 80 mg resmetirom or a placebo once daily. Serial measurements of hepatic fat content were assessed at weeks 12 and 36, and an additional liver biopsy was obtained at week 36 [11]. Of the 348 patients who were screened, 84 received resmetirom, and 41 received a placebo. At week 12 (−32.9% vs. −10.4%; LS mean difference −22.5%, 95% CI −32.9 to −12.2; *p* < 0.0001) and week 36 (−37.3% vs. −8.5%; difference −28.8%, 95% CI −42.0 to −15.7; *p* < 0.0001), hepatic fat was observed to be considerably reduced with resmetirom in patients with evaluable MRI-PDFF. Hepatic fat was reduced by up to 50% relative and 11% absolute in patients with increased medication exposure [11]. These reductions were accompanied by favorable changes in atherogenic lipids (LDL cholesterol, apolipoprotein B, triglycerides, and lipoprotein (a)), as well as in liver function tests, fibrosis markers (PRO-C3, ELF), cytokeratin-18, and adiponectin. Histologic examination indicated resolution of NAS and MASH, particularly in high-exposure groups. Specifically, achieving MASH resolution and ≥1-point fibrosis improvement were highly associated with an MRI-PDFF decrease of ≥30% [11]. The advantages of resmetirom were not influenced by weight loss, which remained modest and consistent across arms. Contrary to its liver-selective thyroid hormone receptor-β activation, the medication was generally well tolerated, with mild, temporary gastrointestinal symptoms (diarrhoea, nausea) being somewhat more common in the treatment group, and no indication of thyroid-related adverse effects [11].

In the same trial, by week 36, resmetirom therapy was linked to notable improvements in physical elements of health-related quality of life (HRQL), namely in physical functioning, body pain, and the Physical Component Summary (PCS) score as determined by the Short Form-36 (SF-36) survey [44]. Patients who benefited the most from the concerned treatment modality were associated with a significant decline in liver fat fraction (LFF) on MRI-PDFF and were also linked with histologic improvements in MASH and fibrosis [44]. The fact that the baseline HRQL scores for the resmetirom and placebo groups were similar demonstrated that these improvements were related to the treatment [44].

### 5.2. Open-Label Extension (OLE) of a Phase 2 Trial (NCT02912260)

In 2021, 36 weeks of open-label extension (OLE) testing were completed to measure how resmetirom affected individuals with nonalcoholic steatohepatitis. Baseline characterization was based on data from 17 individuals using resmetirom in a Phase 2 extension and 14 controls who received placebo in prior studies [43]. For both groups, the treatment continued. Those who had taken resmetirom before were given their usual dose, and others, who had taken a placebo earlier, were given resmetirom at 80 mg. The study group’s average age was 48 years, with 52% being men and 87% of them white, and their average BMI was 35.1 kg/m^2^ [43]. The primary objective was to assess any alteration in the biochemistry of the liver, particularly alanine transaminase (ALT) and aspartate aminotransferase (AST), in those whose levels had not returned to baseline. At week 36 of the extension, the primary effectiveness objective was the relative or absolute change in liver fat fraction (LFF) from baseline as determined by the MRI-proton density fat fraction (MRI-PDFF) [43]. At week 36, the Pla/Res group showed a reduction in LFF of almost 40% (*p* < 0.0001), whereas the Res/Res group showed a drop of 33.5% (*p* < 0.001). Overall, MRI-PDFF decreased by 52.3% when resmetirom (80/100 mg) was used (*p* < 0.0001) [43]. Additionally, improvements in lipid markers were seen, such as decreases in triglycerides (−19.6%), apolipoprotein B (−23.8%), and LDL cholesterol (−26.1%). Fibrosis markers improved, including a decrease in liver stiffness of −2.1 kPa (*p* = 0.015) and a decrease in PRO-C3 levels of −9.8 ng/mL (*p* = 0.0004) [43]. Additionally, the PRO-C3/C3M ratio, which measures the advancement of net fibrosis, was significantly lower. Overall, resmetirom treatment was well tolerated; no severe or major side effects were noted. Resmetirom was well tolerated, supporting its use in the subsequent phase of the MAESTRO-NASH trial [43].

### 5.3. Phase 3 Trials: MAESTRO Program

MAESTRO-NAFLD-1 (NCT04197479) [48] is one of four Phase 3 trials, along with MAESTRO-NASH (NCT03900429) [49], MAESTRO-NAFLD-OLE (NCT04951219) [50], and MAESTRO-NASH-OUTCOMES (NCT05500222) [51], that have been initiated to support an indication for the treatment of MASH with liver fibrosis.

#### 5.3.1. MAESTRO-NAFLD-1 Trial (NCT04197479)

The MAESTRO-NAFLD-1 trial was conducted from 16 December 2019 to 13 December 2021, across 80 sites in the United States. A total of 1988 patients were screened, and 1143 were randomized into the study [45]. The safety and metabolic effects of resmetirom were evaluated in the Phase 3 MAESTRO-NAFLD-1 (NCT04951219) study in 1143 obese individuals with MASLD and suspected MASH who did not qualify for enrolment in MAESTRO-NASH using histologic criteria. In this double-blind, 52-week, randomized, placebo-controlled trial, participants were randomized to receive 80 mg (*n* = 327), 100 mg (*n* = 325), or placebo (*n* = 320). There was also a 100 mg open-label arm [45]. The primary endpoint occurred at similar frequencies across the groups, described as treatment-emergent adverse events (TEAEs), with rates of 86.5% (open-label 100 mg), 86.1% (100 mg), 88.4% (80 mg), and 81.8% (placebo). Diarrhoea and nausea were most frequent at the initiation of treatment [45]. Resmetirom was safe and well-tolerated overall. Hepatic fat content assessed using MRI-PDFF was reduced substantially at 16 weeks (−34.9%, 80 mg; −38.6%, 100 mg) and at 52 weeks (−28.8%, −33.9%) along with a decrease in liver stiffness (−1.02 kPa, −1.70 kPa). Mean percent changes in key secondary lipid endpoints vs. placebo were also significantly improved: LDL-C (−11.1%, −12.6%), apoB (−15.6%, −18.0%), and triglycerides (−15.4%, −20.4%). These findings are consistent with the positive safety profile and therapeutic potential of resmetirom in presumed MASH patients [45].

#### 5.3.2. MAESTRO-NASH (NCT03900429)

Adults with biopsy-confirmed MASH, fibrosis stages F1 to F3, a NAFLD Activity Score (NAS) of 4 or higher, including at least a 1-point score in each of the following components, steatosis, hepatocyte ballooning, and lobular inflammation were recruited for the Phase 3 MAESTRO-NASH trial and were randomly assigned to receive 80 mg, 100 mg, or a placebo once a day [31]. A total of 966 patients were included in the primary analysis population, with 322 receiving 80 mg of resmetirom, 323 receiving 100 mg, and 321 assigned to the placebo group. At 52 weeks, resmetirom also demonstrated a significant advantage over placebo in two co-primary endpoints, including improvement of fibrosis by at least one stage without worsening of MASH (24.2% for 80 mg and 25.9% for 100 mg vs. 14.2% for placebo) and resolution of MASH without worsening of fibrosis (25.9% on 80 mg and 29.9% on 100 mg vs. 9.7% on placebo, all with *p* < 0.001) [31]. Resmetirom also resulted in significant decreases in liver fat fraction (35.4% and 46.6% vs. 8.7% for the placebo) and LDL cholesterol levels (−13.6% and −16.3% vs. 0.1%, *p* < 0.001). Participants also had better liver biochemistry and markers of disease progression. Diarrhoea was reported in 27% to 33.4% of participants treated with resmetirom, compared to 15.6% in the placebo group, with a median duration ranging from 15 to 20 days [31]. Only patients with cirrhosis, other chronic liver diseases, and recent high alcohol consumption were excluded from the trial. These interim results support resmetirom’s potential to alter the disease trajectory in MASH, with ongoing follow-up planned through 54 months to assess long-term outcomes such as progression to cirrhosis and liver-related events [31].

#### 5.3.3. MAESTRO-NAFLD-OLE (NCT04951219)

An extension of the MAESTRO-NAFLD-1 study is assessing the long-term safety and efficacy of resmetirom, with completion expected by April 2026 [48]. The two-year, open-label extension trial data from the MAESTRO-NAFLD-1 study demonstrated the promise of resmetirom in the treatment of compensated MASH cirrhosis [46]. In 101 patients with a baseline mean liver stiffness of 25 kPa (measured by VCTE), the mean decrease was 6.7 kPa [46]. This decrease reached the Baveno VI threshold of 5 kPa and was considered a clinically relevant reduction, indicative of potential fibrosis downstaging. This reduction is the most substantial decrease in liver stiffness reported for an F4c MASH population [46]. In the compensated MASH cohort of MAESTRO-NAFLD-1, resmetirom demonstrated a safety and tolerability profile consistent with previous trials, with a low rate of treatment discontinuation due to adverse events, and no new safety signals were identified [46]. Since MASH cirrhosis is associated with a 42-fold higher risk for liver-related mortality, these results fill another important therapeutic void [46]. Although resmetirom has not yet been approved for the treatment of cirrhosis, the results support further investigation in the ongoing MAESTRO-NASH OUTCOMES trial, which is designed to evaluate clinical outcomes in accordance with FDA guidance5 [46] If successful, resmetirom could become the first approved therapy for F2-F4c MASH, marking a significant advancement in the treatment of progressive liver fibrosis caused by MASH. Until that approval, its use in cirrhotic patients remains investigational [46].

#### 5.3.4. MAESTRO-NASH-OUTCOMES (NCT05500222)

MAESTRO-NASH-Outcome (NCT05500222) is a randomized, double-blind, placebo-controlled, multicentre global study of the safety and efficacy of 80 mg resmetirom in adult patients with well-compensated MASH cirrhosis [51]. Due to be finalized in December 2026, the trial enrols postulated participants with a Child-Pugh A Score (5–6) and a minimum of three metabolic risk factors, with an MRE value of ≥4.2 kPa or an ELF score of >9.8 [51]. Exclusion criteria include other chronic liver diseases, an MELD score greater than 12, and hepatic decompensation. Subjects are randomized 3:1 to resmetirom or placebo, administered orally once daily for approximately three years, following a 60-day screening period [51]. The primary outcome is a composite clinical outcome event, which is a composite of liver-related and cardiovascular mortality/liver transplantation, as well as liver-related events (ascites, hepatic encephalopathy, gastroesophageal variceal haemorrhage, including an increase in MELD from <12 to ≥15). The study aims to determine whether resmetirom can delay or prevent liver-related complications in this high-risk population.

## 6. A Comparative Analysis of Resmetirom and Its Contenders

Resmetirom, a selective thyroid hormone receptor beta (THR-β) agonist, represents a significant milestone in MASH treatment. The MAESTRO-NASH Phase 3 trial achieved meaningful improvements in liver fat content and lipid profiles. These outcomes, combined with a manageable safety profile, underscore its clinical potential as a first-in-class agent for MASH [1,11,31]. A Comparison of efficacy and safety of key agents for MASH has been drawn in Table 2.

Vitamin E has also been investigated as a treatment for MASH, most notably in the PIVENS trial. In this study, high-dose vitamin E (800 IU/day) demonstrated a resolution of MASH in 43% of patients, compared to 19 with placebo (*p* < 0.001) [52]. However, long-term use of vitamin E is associated with adverse effects like an overall increase in all-cause mortality and elevated risk for prostate cancer [53].

Along with these agents, several different drug therapies are currently under study. In a Phase 2, 72-week trial of semaglutide, a GLP-1 agonist, MASH was resolved in more than half (59%) of the patients, whereas only 17% improved in the placebo group (*p* < 0.001) [54]. However, the improvement in the fibrosis was not statistically significant, 43% in patients vs. 33% in placebo (*p* < 0.48). Besides MASH improvement, the drug has also demonstrated significant weight loss and glycaemic control. Adverse events like nausea and vomiting were reported [54]. In a Phase 3 ESSENCE study trial (NCT04822181) [55], a cohort of 1197 patients with biopsy-confirmed MASH and fibrosis stage 2 or 3 were randomized to receive semaglutide 2.4 mg or placebo weekly for 240 weeks. At week 72, semaglutide led to resolution of steatohepatitis without worsening of fibrosis in 62.9% of patients, compared to 34.3% with placebo (*p* < 0.001). Additionally, semaglutide resulted in fibrosis reduction without worsening of steatohepatitis in 36.8% of patients, compared to 22.4% with placebo (*p* < 0.001). Combined resolution and fibrosis reduction occurred in 32.7% versus 16.1% (*p* < 0.001). Mean weight loss was −10.5% with semaglutide vs. −2.0% with placebo. Gastrointestinal side effects were more frequent with semaglutide. Taken together, semaglutide resulted in significant improvements in liver histology in MASH [56].

Pioglitazone, a PPAR-gamma activator, resulted in a primary outcome of ≥2-point reduction in MAFLD activity score in 58% of patients (*p* < 0.001), along with 51% achieving MASH resolution (*p* < 0.001). The trial has also demonstrated improvement in triglyceride levels and improved insulin sensitivity. Weight gain was a notable side effect compared to the placebo [57].

Another potential therapy, obeticholic acid (OCA), is a farnesoid X nuclear receptor agonist initially approved for primary biliary cholangitis [58]. A recent meta-analysis with 2336 participants reviewed the efficacy of OCA in MASH. It revealed significant improvements in liver enzymes, including ALT (MD: −19.48; *p* < 0.05), AST (MD: −9.22; *p* < 0.05), and ALP (MD: 17.61; *p* < 0.05), when compared to MASH patients taking a placebo. It has also revealed a significant reduction in fibrosis (OR: 2.44, *p* = 0.001) and steatosis (OR: 1.82, *p* = 0.001), as well as improvements in lobular inflammation, when compared to patients receiving a placebo. The study revealed no significant differences in adverse effects between the two groups; however, pruritus was noted in the OCA group, especially with higher doses (25 mg: OR 4.72 vs. 10 mg: OR 1.68) [59].

Beyond resmetirom, several other THR-β agonists, such as TG68, CS27109, CS271011, VK2809 (formerly MB07811), and KB141, are currently in early-phase clinical trials for MASH [60]. These drugs have shown promising results in improving lipid profiles and NAS scores, as well as histological improvements. However, further extensive clinical studies are needed to confirm their long-term safety and efficacy [60]. A summary of these investigational agents is provided in Table 3.

Among these, the VOYAGE trial, a 52-week Phase 2b study conducted in 2024, evaluated VK2809 in MASH patients and revealed a significant reduction in liver fat of up to 57% at 12 weeks and 56% at 52 weeks, with a ≥30% reduction achieved in up to 88% of patients. Resolution of MASH without fibrosis was observed in 75% of patients, compared to 29.3% in the placebo population, with the highest efficacy at 10 mg QOD (*p* = 0.0001) [67]. Additionally, patients receiving 5–10 mg also showed ≥1-stage fibrosis improvement without worsening, even in patients with stage 2 or 3 fibrosis. Adverse effects were comparable to placebo [67].

## 7. Safety and Tolerability

The safety and tolerability of resmetirom have been demonstrated in the Phase 3 trials MAESTRO-NASH and MAESTRO-NAFLD-1 [68]. In clinical trials, resmetirom has shown significant improvement in liver enzymes, lipid profile, and MASH biomarkers. It has also demonstrated a notable reduction in LDL-c, triglycerides, lipoproteins, and FT4 levels. Compared to placebo, it has also shown an increase in SHBG and sex steroids. In clinical trials, resmetirom at doses of 80 mg and 100 mg has shown no significant increase in overall treatment-emergent adverse effects (TEAEs) compared to placebo. Specifically, the odds ratio for TEAEs was 1.55 (95% CI: 0.84–2.87) for the 80 mg dose and 1.13 (95% CI: 0.78–1.63) for the 100 mg dose, suggesting no statistically significant difference from the placebo. While the drug is generally well tolerated, gastrointestinal side effects like nausea and diarrhoea have been reported. They were more prevalent in the resmetirom group, particularly within the first 12 weeks of treatment. Diarrhoea occurred in ≥10% of patients receiving resmetirom, with an OR of 3.25 (95% CI: 1.32–7.99; *p* = 0.01) for events occurring before 12 weeks. Nausea also occurred in ≥10% of patients, with an OR of 1.98 (95% CI: 1.09–3.62; *p* = 0.03) for events within the same timeframe [2,69,70]. These symptoms were typically seen within the first 12 weeks of treatment, with a median duration of 15–20 days. However, these side effects are generally mild and transient [71].

Liver function test abnormalities have also been reported. Within the first 4 weeks of treatment, mild, transient elevations in aminotransferases developed in a significant proportion of people. However, they were self-limited and were not associated with any symptoms or jaundice, often returning to baseline within 3–6 months [72]. A case of significant hepatotoxicity has been reported where one patient developed significant liver injury with jaundice, which resolved upon discontinuation of resmetirom. Upon re-evaluation, the patient was found to have met the criteria for primary biliary cholangitis. This was supported by strongly positive anti-mitochondrial and anti-nuclear antibodies at baseline. The adjudication committee concluded that there was a 25–50% chance that resmetirom could have led to this condition [73]. Such cases underscore the importance of regular liver function monitoring during the therapy. Gall bladder-related adverse events such as cholelithiasis, acute cholecystitis, and obstructive pancreatitis have been reported to occur more often in the resmetirom group compared to the placebo group in clinical trials [74].

The MAESTRO-NASH trial showed no significant difference in the incidence of serious adverse events across all trial groups: 10.9% in the 80 mg resmetirom group, 12.7% in the 100 mg resmetirom group, and 11.5% in the placebo group. According to a study by Ravela et al., the common reasons for patients discontinuing Resmetirom are its side effects, namely diarrhoea, nausea, abdominal pain, rash, pruritis, and dizziness. In both trials, resmetirom showed no increase in major cardiovascular events, bone fractures, changes in BMD, endocrine issues, or thyroid-related symptoms compared to placebo. This further underscores the importance of monitoring and managing side effects in real-world clinical practice [75].

The favorable safety profile of resmetirom has also been confirmed by the safety data from the MAESTRO-NAFLD study, which included more than 1100 patients treated for 52 weeks. Currently, 700 patients are scheduled to be enrolled in the MAESTRO-NASH OUTCOMES (time-to-event) study, which is anticipated to last two to three years. It will also provide additional information on the long-term safety and efficacy of resmetirom by evaluating the time to composite clinical outcome events, including all-cause mortality, liver transplantation, hepatic decompensation (ascites, hepatic encephalopathy, gastrovariceal haemorrhage), hepatocellular carcinoma, or an increase in MELD score from <12 to ≥15. In addition, whether resmetirom can delay or halt disease progression in patients with well-compensated MASH-cirrhosis will be assessed.

The safety of resmetirom is acceptable according to the MAESTRO-NAFLD-1 and MAESTRO-NASH studies; however, it should be used with caution in certain populations. It should not be used in patients with decompensated cirrhosis, and it may interact with statins, fibrates, clopidogrel, and cyclosporine, causing a dose adjustment to be necessary [29]. It has not been tested for safety and effectiveness in individuals under 18. Resmetirom activates thyroid hormone, thereby stimulating intracellular energy expenditure that results in mitochondrial beta-oxidation of fatty acids, creating reactive oxygen species (ROS) as a by-product [76]. The long-term impact of elevated ROS levels in hepatocytes is still unclear.

## 8. Limitations of Current Evidence

The existing data suggests that resmetirom is effective and safe for MASH; nevertheless, there are significant limitations that must be noted. The bulk of our data comes from highly controlled clinical trial populations, which may not completely reflect the variety of real-world people, many of whom have numerous comorbidities, use different medicines, and follow lifestyle changes to variable degrees. Second, most clinical studies thus far had short follow-up periods, making it difficult to determine how resmetirom impacts liver-related and cardiovascular outcomes, progression to cirrhosis, and overall survival in the long run. Third, although surrogate endpoints such as liver fat reduction, histologic improvement, and biomarker changes have shown promise, there is still a lack of significant outcome data, such as a reduction in liver-related mortality, the need for transplantation, or cardiovascular events. Furthermore, differences in payer criteria and access limitations may limit the generalizability and equitable implementation of these results across different healthcare systems. All of these constraints highlight the need for long-term, practical research and collecting real-world data to ensure that resmetirom treatment is effective over time and in a broader variety of circumstances.

## 9. Future Direction

The approval of resmetirom marks a pivotal shift in the management of MASH, a hepatic manifestation of metabolic syndrome that often coexists with T2D, obesity, hypertension, and dyslipidaemia [77]. Cardiovascular disease is still the most common cause of death in MASH patients, but it is largely unaddressed. Resmetirom has demonstrated efficacy in reducing hepatic fat and fibrosis and is recommended according to AASLD guidelines. Its best use is combined with non-pharmaceutical interventions (NPIs), such as diet, physical activity, and psychological support, to improve the patient’s overall wellness. However, disparities between clinical trial inclusion criteria and real-world payer criteria, such as those from the Veterans Administration, highlight access barriers and underscore the need for policy-level solutions to prevent the widening of healthcare inequities.

The use of resmetirom also requires structured CME programs to prepare hepatologists, endocrinologists, and primary care clinicians to perform active case finding as well as multidisciplinary care [77]. A disconnect remains in international screening and fibrosis diagnosis algorithms, highlighting the need for harmonized, actionable approaches. Future studies should aim to assess the real-world use of resmetirom, particularly in hepatic-beneficial agents, including GLP-1 receptor agonists, SGLT2 inhibitors, and statins, in more general populations. Statins, in particular, may offer additive antifibrotic and anti-inflammatory effects via inhibition of the NLRP3 inflammasome and require dedicated combinatorial studies with resmetirom.

MASH pathogenesis is multifactorial, with genetic polymorphisms (e.g., PNPLA3, TM6SF2), metabolic stress, and disrupted thyroid hormone signalling all contributing [39]. While trials did not stratify by genotype, the benefits of resmetirom were consistent across populations, particularly among those with obesity. Long-term follow-up is necessary to evaluate its effect on the hypothalamic–pituitary–thyroid axis and its safety in patients with thyroid diseases.

The question of fair access relies on clear payer endpoints, QALY estimates that account for disease onset in younger children, and cost-effectiveness data. Finally, robust patient-reported outcomes will be crucial for evaluating both the real-world impact and treatment acceptability, as well as for informing future regulatory and reimbursement decisions. The introduction of resmetirom, concomitant with a systematic overhaul and implementation grounded in evidence of NPIs, offers a paradigm-shifting prospect in MASH care.

## 10. Conclusions

Resmetirom has emerged as a promising therapy for metabolic dysfunction-associated steatohepatitis (MASH). It preferentially activates thyroid hormone receptor-β, making it more effective for certain individuals. This thorough study demonstrates that it may reduce hepatic steatosis, improve lipid profiles, and perhaps halt the course of fibrosis, all while maintaining a high safety profile. Although early trials indicate promising outcomes, we need additional data from Phase 3 research to determine the long-term benefits and how it fits into treatment recommendations. As MASH grows increasingly frequent across the world and there are few treatment choices, resmetirom provides a novel technique to alter the course of the condition that might be highly beneficial.

## Figures and Tables

**Table 1:** Summary of clinical trials evaluating the efficacy and safety of resmetirom in patients with MASH.

Feature	Phase 2 [11] (NCT02912260)	Phase 2 OLE [43] (NCT02912260)	Phase 2 HRQL [44] (NCT02912260)	MAESTRO- NAFLD-1 [45] (NCT04197479)	MAESTRO- NAFLD-OLE [46] (NCT04951219) 2 Years of Data	MAESTRO- NASH [31] (NCT03900429)
**Study Type**	Phase 2, DB, RCT	OLE (36-week extension)	Phase 2, HRQL focused	Phase 3, DB, RCT	Phase 3, OLE of MAESTRO-NAFLD-1	Phase 3, DB, RCT
**Population**	125 biopsy-proven MASH (F1–F3), ≥10% hepatic fat	31 patients (14 former placebo), elevated LFTs	125 MASH, assessed for HRQL	1143 NAFLD with presumed MASH	101 patients with compensated MASH cirrhosis (F4c)	966 biopsy-proven MASH with F1B–F3
**Doses**	80 mg vs. placebo	80 or 100 mg (open-label)	80 mg vs. placebo	80 mg, 100 mg, placebo	100 mg (open label)	80 mg, 100 mg, placebo
**Duration**	36 weeks	36 weeks	36 weeks	52 weeks	2 years	52 weeks
**Primary Endpoints**	MRI-PDFF% % fat reduction at week 12	MRI-PDFF change at week 36	HRQL (SF-36) improvement	Safety (TEAEs)	Reduction in liver stiffness measured by VCTE	MASH resolution and fibrosis improvement
**Key Outcomes**	MRI-PDFF ↓32.9% at week 12 (vs 10.4% placebo); *p* < 0.0001	MRI-PDFF ↓52.3%; LDL ↓26.1%; ApoB ↓23.8%; Trigs ↓19.6%; Fibrosis markers improved	Physical scores and SF-6D improved with ≥30% fat reduction	Hepatic fat ↓ up to 38.6%; LDL, ApoB, Trigs ↓; liver stiffness ↓	Mean liver stiffness reduced from 25 kPa at baseline to 18.3 kPa at 2 years (6.7 kPa reduction) 51% of patients had ≥25% improvement in liver stiffness; the largest reported liver stiffness reduction in the F4c MASH population to date Mean reduction exceeded the ‘Baveno rule of 5 kPa’ threshold for lower risk stratification	MASH resolution: 25.9–29.9% vs. 9.7% (placebo); fibrosis ↓ ≥1 stage: 24.2–25.9% vs. 14.2%
**Safety**	Mild/moderate AEs: diarrhoea and nausea ↑	Well tolerated; few non-serious AEs	No serious AE	Safe and well tolerated; diarrhoea/nausea at initiation	Consistent safety and tolerability profile with other trials	Similar AE incidence across groups
**Conclusion**	Effective in reducing hepatic fat at 12 and 36 weeks	Supports efficacy, safety, and noninvasive monitoring	Supports efficacy, safety, and noninvasive monitoring	Fat loss is linked to improved quality of life	Promising efficacy in reducing liver stiffness in compensated MASH cirrhosis	Both doses were superior to placebo in MASH resolution and fibrosis improvement

**Note: DB:** Double Blind. **RCT:** Randomized Controlled Trial **AE:** Adverse Event.

**Table 2:** Comparison of efficacy and safety of key agents for MASH.

Agent	Fibrosis Regression (vs. Placebo)	MASH Resolution (vs. Placebo)	Key Safety Concerns	References
**Resmetirom**	Moderate (better than placebo)	Moderate (better than placebo)	Generally well tolerated; mild/moderate GI side effects (diarrhoea, nausea)	[1]
**Semaglutide (GLP-1)**	Moderate	Modest to moderate	GI side effects, weight loss	[2]
**Vitamin E**	Modest, best in non-diabetics	Modest	Long-term safety unclear	[2]
**Pioglitazone**	Moderate to Robust (especially with vitamin E)	Moderate to robust	Weight gain, oedema, heart failure	[2]
**Obeticholic Acid**	Modest to moderate	Moderate	Pruritus, ↑ LDL, long-term safety concerns	[2]

**Table 3:** THR-β agonists’ preclinical efficacy against MASH in animal models.

Year and Study	Model Used	Dose & Duration	Key Effects Observed
**Polini et al., 2025** **(TG68)** [61]	HFD-fed mice	10 mg/kg/day × 2 weeks (oral)	↓ Body weight (12%) ↓ Lipids and glucose ↑ THRβ-target genes (DIO1, DIO3) ↓ HMG-CoA reductase
**Caddeo et al., 2023** **(TG68)** [62]	HFD-fed rats	9.35 mg/kg × 3 weeks (oral)	↓ Liver weight and LW/BW ratio ↓ TGs, CH, glucose ↑ Cholangiocyte proliferation
**Huang et al., 2023** **(CS27109)** [63]	HFD mice	10–30 mg/kg × 4 weeks	↓ TC, LDL-c, AST (dose dependent) ↓ ALT only at high dose ↓ Liver steatosis ↓ NAS score
**Lin et al., 2023** **(CS271011)** [64]	DIO mice	1–3 mg/kg × 10 weeks	↓ TC, TG (dose dependent) ↓ Steatosis, ballooning ↓ NAS score No ALT/AST elevation
**Erion et al., 2007** **(MB07811)** [65]	DIO mice, SD rats	Up to 30 mg/kg/day × 2 weeks	↓ TC (61%), TG (40%) ↓ Liver TGs, liver weight BW loss (3–4%)
**Grover et al., 2003** **(KB-141)** [66]	TRα1−/− and WT mice	Various doses	↓ Weight, TC, Lp(a) ↑ Heart weight ↓ T3/T4 (strong suppression)

**Note: THR-β:** Thyroid Hormone Receptor Beta. **TG**: Triglycerides. **LDL-C**: Low-Density Lipoprotein Cholesterol. **HDL-C**: High-Density Lipoprotein Cholesterol. **ALT**: Alanine Aminotransferase. **AST**: Aspartate Aminotransferase. **DIO**: Diet-Induced Obesity. **HFD**: High-Fat Diet. **LDLR**: Low-Density Lipoprotein Receptor. **TC**: Total Cholesterol. **FFA**: Free Fatty Acids. **HMG-CoA**: 3-Hydroxy-3-Methylglutaryl-Coenzyme A. **TRα1−/−**: Thyroid Hormone Receptor Alpha 1. **WT**: Wild Type.

## Data Availability

No new data were created or analyzed in this study. Data sharing is not applicable to this article.
